# Electrical, Hemodynamic, and Motor Activity in BCI Post-stroke Rehabilitation: Clinical Case Study

**DOI:** 10.3389/fneur.2018.01135

**Published:** 2018-12-20

**Authors:** Alexander A. Frolov, Pavel D. Bobrov, Elena V. Biryukova, Anna V. Silchenko, Anna A. Kondur, Indiko Z. Dzhalagoniya, Jean Massion

**Affiliations:** ^1^Research Institute of Translational Medicine, Pirogov Russian National Research Medical University, Moscow, Russia; ^2^Laboratory of Mathematical Neurobiology of Learning of Institute of Higher Nervous Activity and Neurophysiology, Russian Academy of Science, Moscow, Russia; ^3^Faculty of Physics, Moscow State University, Moscow, Russia; ^4^Department of Neurology, Vladimirsky Moscow Regional Research Clinical Institute, Moscow, Russia; ^5^CNRS, Marseille, France

**Keywords:** brain-computer interface, rehabilitation, stroke, inverse problem in EEG source localization, functional magnetic resonance imaging, biomechanics

## Abstract

The goal of the paper is to present an example of integrated analysis of electrical, hemodynamic, and motor activity accompanying the motor function recovery in a post-stroke patient having an extensive cortical lesion. The patient underwent a course of neurorehabilitation assisted with the hand exoskeleton controlled by brain-computer interface based on kinesthetic motor imagery. The BCI classifier was based on discriminating covariance matrices of EEG corresponding to motor imagery. The clinical data from three successive 2 weeks hospitalizations with 4 and 8 month intervals, respectively were under analysis. The rehabilitation outcome was measured by Fugl-Meyer scale and biomechanical analysis. Both measures indicate prominent improvement of the motor function of the paretic arm after each hospitalization. The analysis of brain activity resulted in three main findings. First, the sources of EEG activity in the intact brain areas, most specific to motor imagery, were similar to the patterns we observed earlier in both healthy subjects and post-stroke patients with mild subcortical lesions. Second, two sources of task-specific activity were localized in primary somatosensory areas near the lesion edge. The sources exhibit independent mu-rhythm activity with the peak frequency significantly lower than that of mu-rhythm in healthy subjects. The peculiarities of the detected source activity underlie changes in EEG covariance matrices during motor imagery, thus serving as the BCI biomarkers. Third, the fMRI data processing showed significant reduction in size of areas activated during the paretic hand movement imagery and increase for those activated during the intact hand movement imagery, shifting the activations to the same level. This might be regarded as the general index of the motor recovery. We conclude that the integrated analysis of EEG, fMRI, and motor activity allows to account for the reorganization of different levels of the motor system and to provide a comprehensive basis for adequate assessment of the BCI+ exoskeleton rehabilitation efficiency.

## Introduction

The methods of post-stroke rehabilitation using the limb exoskeleton controlled by brain-computer interface (BCI) based on kinesthetic motor imagery (MI) may be helpful for the motor function recovery (1). The procedure efficiency has been shown by several randomized controlled studies (2–8). Yet case studies of patients involved in BCI procedures are not numerous. The studies mainly concern the BCI system design and improvement (9–11) while smaller number of them are centered on different aspects of the procedure outcome, both motor (12) and psychological (13).

The present study is aimed to demonstrate the results of integrated analysis of electrical, hemodynamic, and motor activity accompanying the motor function recovery. The results were obtained for a patient with an extensive post-stroke cortical and subcortical lesion in a chronic post-stroke period. There were two major reasons for selecting the patient for the study. First, she demonstrated high accuracy of BCI control. This suggests high contrast and stability of her EEG patterns specific to motor imagery, making identification of these patterns easier. Second, she went through three hospitalizations, which allowed us to investigate the long-term treatment effects.

The motor outcome of BCI+ hand exoskeleton rehabilitation was assessed both using the FM score (14) and biomechanical analysis of upper limb movements before and after the interventions. The biomechanical analysis was used to avoid possible subjectivity of the FM scoring and, what is more crucial, to capture the motor function tiniest alterations as they might testify to degree of motor recovery (15).

The EEG analysis was based on the previously developed methodology which was applied to find sources of electrophysiological brain activity the most specific for motor imagery during the BCI control by both healthy subjects and post-stroke patients with mild subcortical lesions (16–18). The hemodynamic activity was investigated through the comparative analysis of fMRI data before and after the course of rehabilitation during the final hospitalization period.

## Methods

### Patient

The patient, female, 42 years old, was recruited in the study 16 months after hemorrhagic stroke. Following the MRI data the lesion was located in cortical-subcortical areas of the frontal lobe in the left hemisphere (Figure 3A). The muscle tone and tendon reflexes of the paretic arm were increased. The patient was able to follow the instructions of the rehabilitation procedure (the score of Montreal Cognitive Assessment amounted to 26) and had no other neurologic, neuromuscular or orthopedic diseases. The muscle force was assessed by Medical Research Council (MRC) scale as 1 for distal domain of the arm and as three for proximal one during all the three hospitalizations. Anxiety and depression were assessed by Hospital Anxiety and Depression Scale (HADS) (19) as 4/4 for first and second hospitalizations which corresponds to the norm (< 7) and as 8/7 for the third one, which corresponds to subclinical anxiety/depression. The patient went in for sports and was familiar with kinesthetic motor imagery. In addition, the patient was highly motivated for rehabilitation, which stimulates the efficiency of BCI-based procedures (20, 21). The patient met the inclusion criteria elaborated for the BCI+ Exoskeleton clinical trials (8).

The patient went through three 2-weeks hospitalizations with 4 and 8 months intervals, respectively. She was provided with standard therapy in accordance with Russian treatment protocols and standards. Each hospitalization the therapy was complemented with 10 BCI+ Exoskeleton sessions, one session a day.

### BCI+ Exoskeleton Procedure

**Figure 1:**
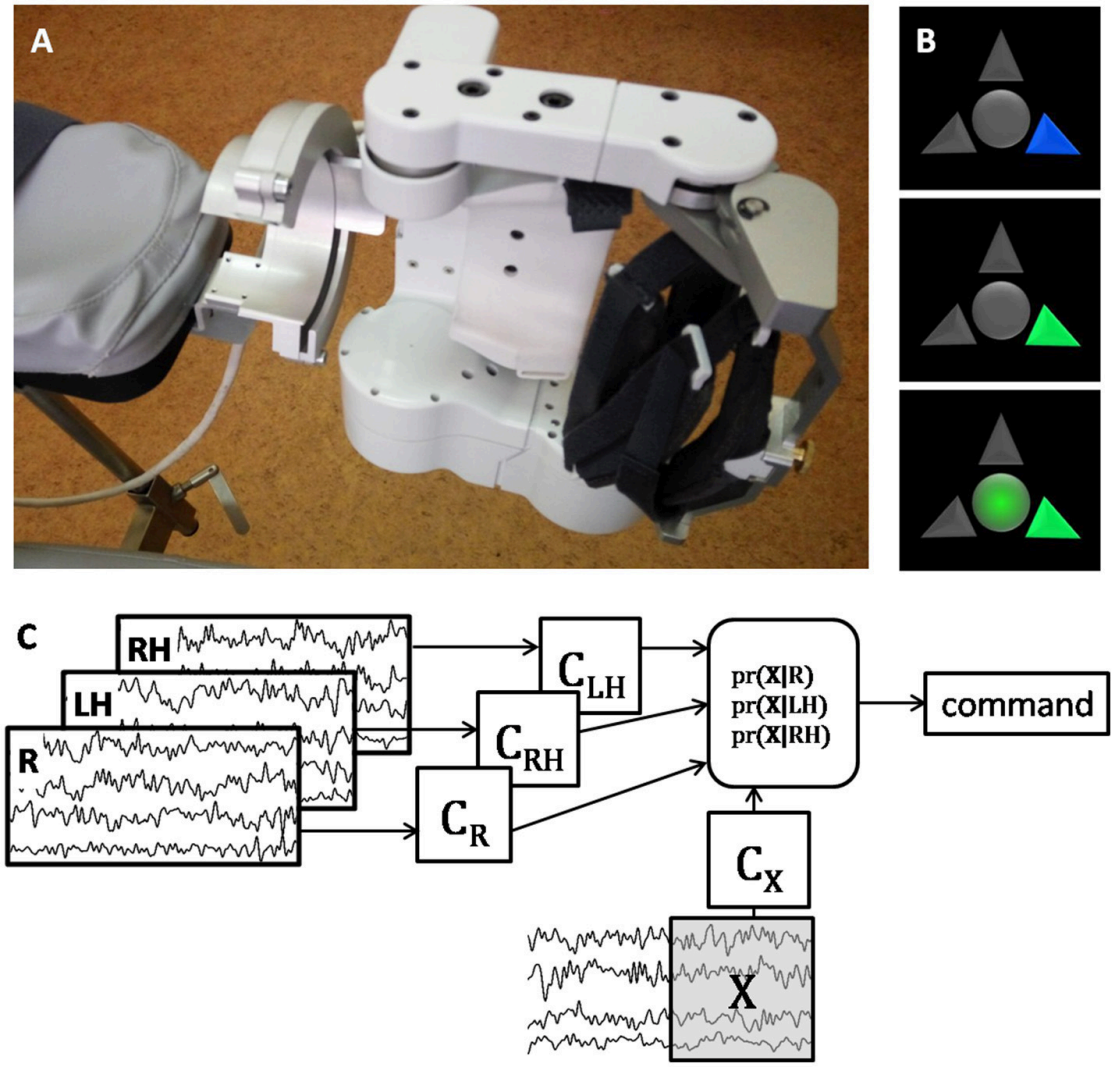
**(A)** photo of the left exoskeleton attached to the chair arm pad; the right exoskeleton has symmetrical construction. **(B)** visual cues of the BCI system; the upper arrow corresponds to relaxation, the left and right arrows correspond to the left and right hand extension imagery; the blue arrow signals the patient to prepare for performing the corresponding task; the green arrow signals the patient to start performing the task; circle in the middle of the screen serves both as a gaze fixation point and for additional visual feedback, it becomes green when the BCI classifier recognizes that the cued task is executed. **(C)** schematic illustration of the BCI classification algorithm. After several presentations of each task the corresponding EEG covariance matrices (C_R_ for Relaxation, C_LH_ and C_RH_ for the left and hand motor imagery) are estimated. Then for each sliding 1 s EEG window, X, the probability of its corresponding to each task is estimated using the Bayesian approach. The command is generated based on the maximal probability, and the window is shifted as new EEG data are acquired. The more correct answers the BCI provides, the more is the exoskeleton extended.

During the BCI treatment the patient was sitting in a medical chair with her hands placed into two exoskeletons (Android Technics, Russia). She controlled extension of two hand exoskeletons (Figure 1A) by performing motor imagery tasks, following visual cues presented on the monitor (Figure 1B). The tasks were to relax (R) and to imagine kinesthetically slow extension of either the paretic (right, RH) or the intact (left, LH) hand fingers. The exoskeleton extended the patient's fingers if the BCI classifier recognized the imagery of their extension.

The EEG data were recorded with NVX52 device (Medical Computer Systems, Russia) with 40 AgCl electrodes. The data were digitized at 500 Hz, filtered on-line with band-pass 5–30 and 50 Hz notch filters, and fed to the BCI classifier. The raw EEG data were stored for further processing.

Each day electrode positions on the scalp were digitized with the EEG PinPoint system (Localite GmbH, Germany) after the daily BCI session was over. The positions were set in the MRI anatomical image coordinate system.

The BCI classification algorithm was designed under the assumption that EEG has a multivariate Gaussian distribution with zero mean and covariance matrix depending on the task performed (22). The BCI classified ongoing EEG signal by comparing its covariance matrix obtained for the last second epoch with the three EEG covariance matrices corresponding to the R, LH, and RH tasks (Figure 1C). The details of the experimental setup and procedure are given in (8).

### Motor Function Assessment

The motor functions were assessed using the standard clinical FM score and biomechanical analysis of upper limb movements. The movements were registered by the TrakStar electromagnetic system (Ascension Technology Corp., USA). Four sensors were placed on the patient's hand, forearm, upper arm and acromion. Their positions and orientations were digitized at 100 Hz. Passive and active movements along each of the seven arm degrees of freedom (DoF) were recorded to calculate the individual joint axes and the rotation angles in the joints (23). FM assessment and motion recordings were performed at the beginning and at the end of each hospitalization for both intact and paretic arm. Mean absolute values of all angular velocities (MAV), considered as indices of muscle forces (24) were computed for each DoF.

### *Post-hoc* EEG Analysis

For the sake of *post-hoc* analysis the raw data were filtered with 5–30 Hz band-pass zero-phase FIR filter and 50 Hz zero-phase notch filter to avoid power line interference. The filtered data of each session were decomposed into components using several independent component analysis algorithms: AMICA (25), FastICA (26), RunICA (27), kurtosis optimization (28), PWCICA (29), and variance non-stationarity maximization (30). Each component is defined by its temporal activity and voltage distribution over EEG electrodes (topographic map), resulting from a certain source of electrophysiological activity. Since the sources which may be related to the EEG activity prove to be dipolar (31, 32), only components which distributions could be accurately approximated with that of a single dipolar source were selected for further analysis. The approximation was considered accurate if the residual variance of a single dipole fit did not exceed 5%. The dipole was fitted using individual head model created from T1- and T2-weighted MRI images and the digitized electrode positions. For model creation and fit technique see our previous works (16, 18).

The components selected from all experimental sessions were grouped into clusters according to their topographic maps similarity. The clustering was performed using the Attractor Neural Network with Increasing Activity (33, 34). We assume that finding a component by several ICA methods with different criteria of component independency makes it more certain that the component actually corresponds to the specific source of brain activity. Thus, in case two or more components of the same session were assigned to the same cluster, only the component found by more methods was chosen for further analysis. The components found by different methods for the data of the same session were considered to be identical if the cosine between the component distribution vectors (the topographic maps) exceeded 0.95 and the correlation coefficient between their activities exceeded 0.8. The component relevance for the BCI performance was estimated using the algorithm described in (16). Current source density of the relevant components was estimated by sLORETA (35) using the individual head model and the digitized electrode positions.

### MRI and fMRI Registration and Analysis

Scanning was performed on a 3T Siemens Magnetom Verio at the beginning and at the end of third hospitalization. The anatomical images were acquired using T1-weighted multiplanar reconstruction mode (TR = 1,900 ms, TE = 2.47 ms, 512 × 512 matrix, FOV = 250 × 250 mm) with 0.488 × 0.488 × 1 mm voxel size.

The fMRI data were acquired with BOLD sensitive T2^*^-weighted mode (36 slices, TR = 3,000 ms, TE = 30 ms, 64 × 64 matrix, FOV = 192 × 192 mm, BW = 2,232 Hz/pixel) with 3 × 3 × 3 mm voxel size. 120 3D images were obtained during each session (one session before and one after training). Experimental design contained consequent trials of the mental tasks used to control BCI. Each trial lasted 30 s and contained 10 full brain scans.

The fMRI data were processed according to SPM12 (Statistical Parametric Mapping, Wellcome Trust Centre for Neuroimaging at UCL, London, UK) standard single subject processing workflow. Two *t*-test contrasts were used to identify areas where activation during left and right hand MI was significantly different from activation during relaxation (*p* < 0.001).

## Results

### Accuracy of the BCI Control

The average probabilities of the correct BCI classification were 0.61 ± 0.02 (max. 0.77), 0.58 ± 0.015 (max. 0.75) and 0.62 ± 0.02 (max. 0.84) for the first, second, and third hospitalization, respectively. There was no significant difference between the BCI control accuracy for different hospitalizations (*p* > 0.17, pair-wise Wilcoxon test). There were also no evident trends in the classification accuracy during each hospitalization.

### Motor Output

The total FM score increased from 75 to 85 (from 4 to 8 in the distal and from 17 to 20 in proximal domains), from 74 to 89 (from 3 to 7 in the distal and from 22 to 24 in the proximal domains), and from 75 to 84 (from 3 to 5 in the distal and from 22 to 24 in the proximal domains) during the first, second, and third hospitalizations, respectively. Thus, for each hospitalization the improvement of motor functions exceeded five points, the minimal clinically important difference for chronic patients (36, 37).

Wrist DoFs and pronation-supination of the forearm were the most affected. Biomechanical analysis showed increasing of the forearm pronation-supination MAV in both the paretic and intact arms. During the first hospitalization the MAV increased from 0.06 to 0.12 rad/s for the paretic arm and from 2.95 to 4.95 rad/s for the intact arm. Corresponding MAV values increased from 0.09 to 0.24 rad/s and from 4.7 to 5.2 rad/s during the second hospitalization, and from 0.07 to 0.11 rad/s and from 4.47 to 4.68 rad/s during the third one. The MAV of the wrist DoFs never exceed 0.06 rad/s.

### BCI Biomarkers

**Figure 2:**
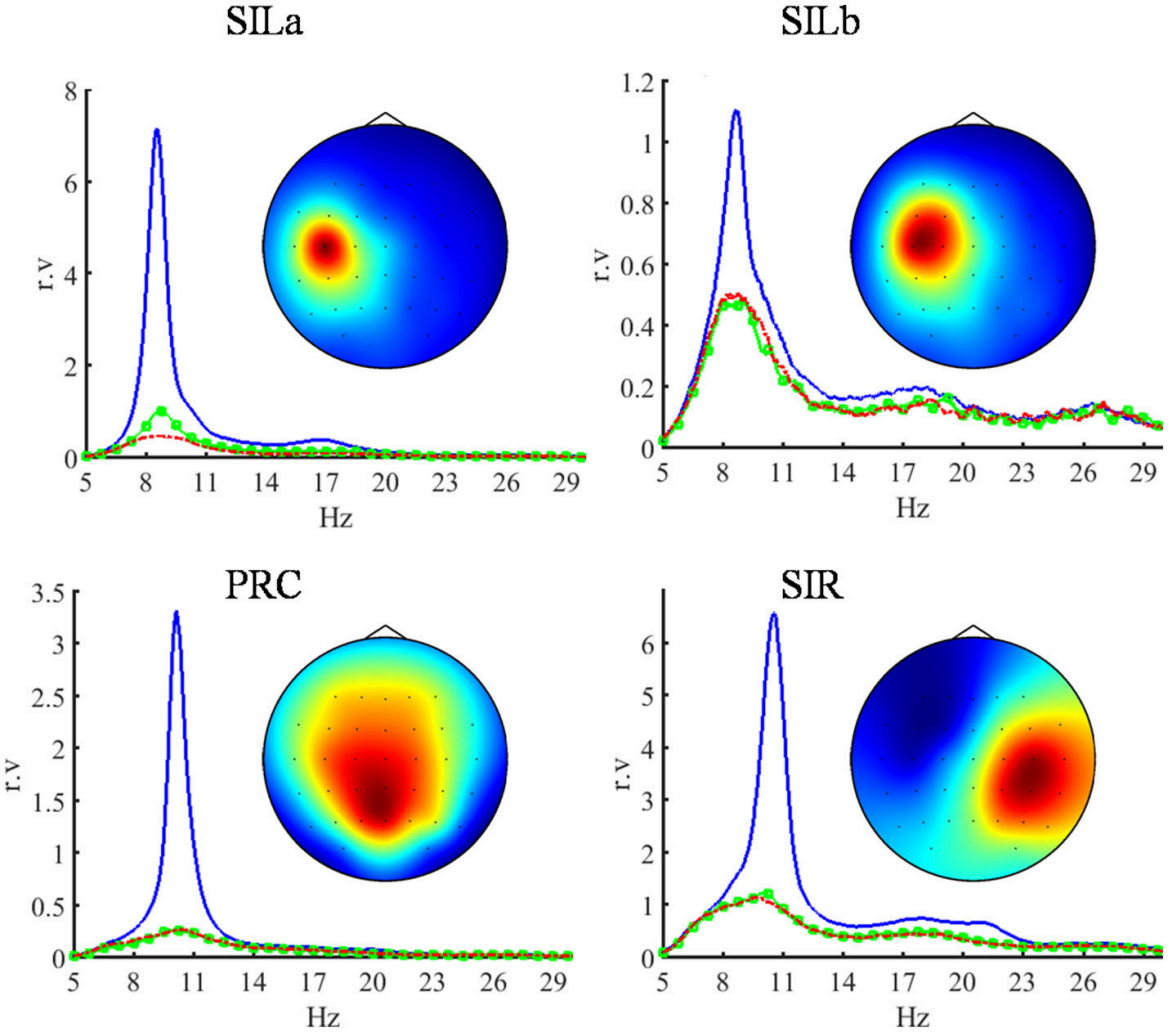
Topographic maps and power spectral densities (PSD) of the four most relevant independent EEG components which are the main BCI biomarkers. The sources of their activity are located in the left primary somatosensory area (SILa, SILb), in the precuneus (PRC) and in the right primary somatosensory area (SIR). The power spectral densities are shown in 5–30 Hz band. The blue lines indicate relaxation, the green and red lines indicate the imagery of the left (intact) and right (paretic) hand extension, respectively. The PSD of each component is given in relative units proportional to V^2^/Hz, since the component activity scale is undefined (28). The components exhibit the suppression of EEG rhythmic activity during the motor imagery. The sources located in the damaged hemisphere have lower peak frequency compared to the sources in the intact areas.

**Figure 3:**
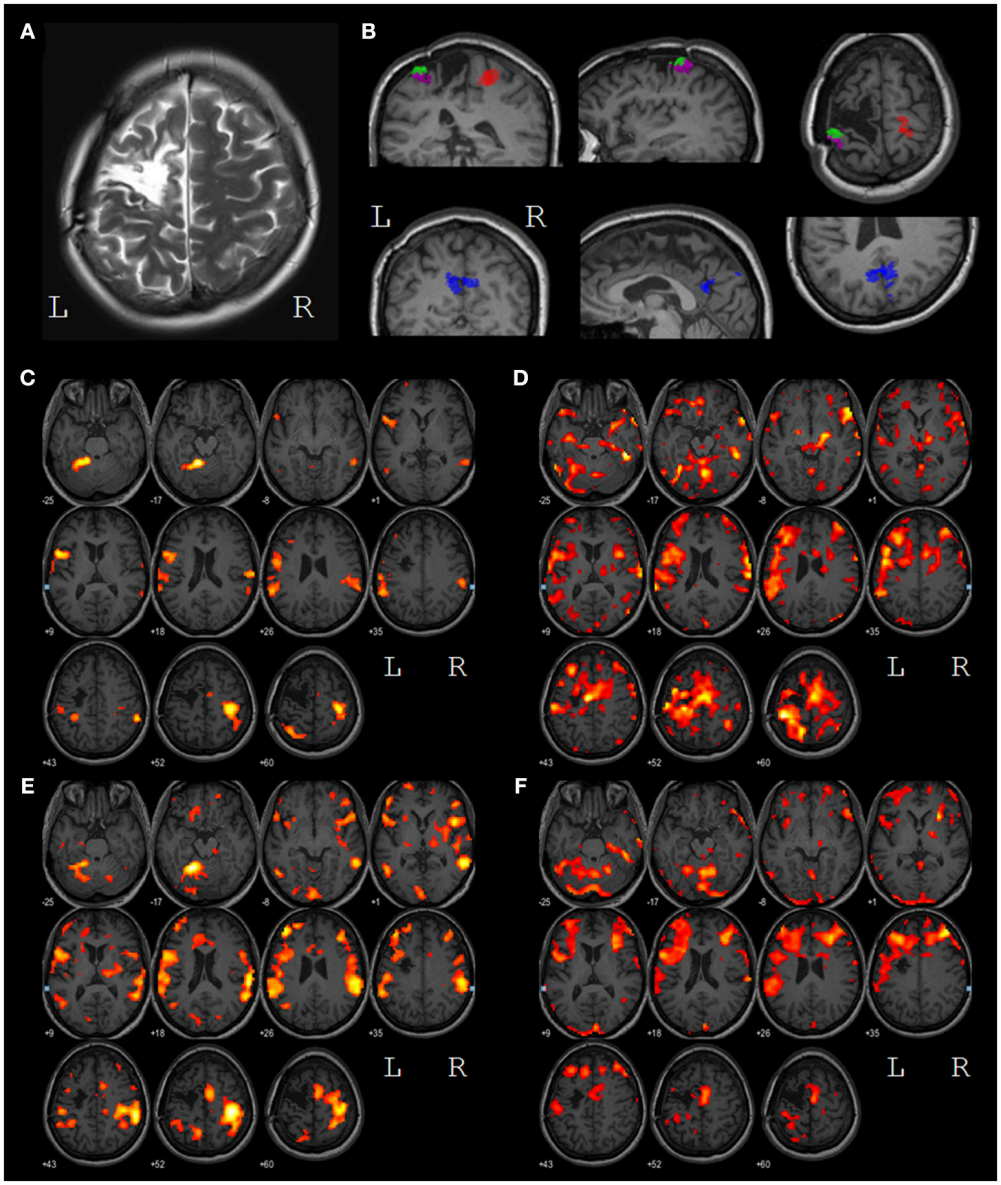
**(A)** T2 contrasted image of the lesion in the left motor and premotor areas; **(B)** results of the task-specific EEG source localization. The SIR source (red) is located in the primary somatosensory area of the right (intact) hemisphere, the SILa,b sources (green and magenta) are located in the primary somatosensory area of the left hemisphere, near the lesion edge, the PRC source (blue) is located in the precuneus. **(C)** voxels with BOLD response significantly higher during LH MI than during relaxation (*p* < 0.001) *before* the treatment; **(D)** voxels with BOLD response significantly higher during RH MI than during relaxation (*p* < 0.001) *before* the treatment; **(E)** voxels with BOLD response significantly higher during LH MI than during relaxation (*p* < 0.001) *after* the treatment; **(F)** voxels with BOLD response significantly higher during RH MI than during relaxation (*p* < 0.001) *after* the treatment. All images are presented in the neurological convention. The left and the right hemispheres are marked by L and R, respectively.

The data of 72 EEG recordings were processed, 24 recordings for each hospitalization. The component clusters were sorted according to the number of elements. The components of each of the first four clusters appeared in more than 65% sessions while the components of all other clusters appeared in < 20% sessions. Moreover, the components of each of the four clusters were relevant for the BCI performance in more than 50% of sessions while the components of all the rest clusters were relevant in < 40% of sessions. The component relevance means that changes in their activity are the main cause for the difference between the task-specific EEG covariance matrices. That is why the components of the first four clusters, which were the most common and relevant, were considered as the main BCI biomarkers and were selected for detailed analysis. The topographic maps (Figure 2), activity spectral densities (Figure 2), and source locations (Figure 3B) suggest that components of the selected clusters represent the activity of the primary somatosensory areas of the left (SILa, SILb) and right (SIR) hemispheres and precuneus (PRC). The components demonstrate the suppression of alpha-band EEG activity during the motor imagery. The peak frequencies of SIR and PRC sources located in the intact areas were 10.54 and 10.29 Hz, while the peak frequencies of the SILa and SILb sources located near the lesion were significantly lower: 8.69 and 8.54 Hz (*p* < 0.001, Wilcoxon test). The SILb sources were always found together with SILa although less frequently (66% sessions vs. 94% sessions). Despite the proximity of SILa and SILb locations the sources exhibit independent activity.

The SILa, SILb, SIR, and PRC source occurrence and relevancy rates were consistent for all hospitalizations, as well as their locations and peak frequencies.

### Hemodynamic Activity

Both before and after the treatment, the primary sensory-motor cortex, and the supplementary motor area (SMA) of the right hemisphere, the supramarginal gyri of both hemishperes and areas BA44/45 of the left hemisphere (Broca area) were activated during the intact (left) hand MI (Figures 3C,E). After the treatment the volume of all the areas of activity increased with the only exception for a zone in the upper parietal lobe.

During the paretic (right) hand MI, the activity before the treatment was represented by the extensive clusters in the primary sensory-motor areas, in the SMA, in the frontal lobes, in the temporal gyrus and in the cerebellum of both hemispheres (Figures 3D,F). After the treatment areas of activation decreased in size. The overall levels of activation during intact and paretic hand MI shifted to the same level.

## Discussion

In spite of the extensive post-stroke cortical and subcortical lesion the patient was able to control the BCI as accurately as the best of healthy subjects (18). Along with high motivation it testifies to the favorable rehabilitation potential (38). The accuracy of BCI control did not exhibit significant trends during the BCI training in all three hospitalizations, while FM score and biomechanical indices of the paretic arm recovery increased. Also, biomechanical analysis revealed motor improvement for the intact arm. The motor improvement for both arms may result from the patient's high ability for motor imagery.

At the beginning of every following hospitalization the FM scores were lower than at the end of the previous one. This suggests that while the BCI training is efficient for the motor recovery, the improvement in the motor function may not remain at the level attained after 2 weeks long treatment. The long-term positive effect of the BCI training manifested in the higher indices of the motor recovery during the second hospitalization as compared to the first one.

During the imagery of the left (intact) hand areas of fMRI activations were almost the same as for healthy subjects (39) with the predominance of the right hemisphere activity. During the imagery of the paretic hand large clusters of fMRI activity were observed in both hemispheres. After BCI training the areas of fMRI activity expanded during the intact hand motor imagery and shrank for the paretic hand. The reduction of fMRI activity foci during the paretic hand motor imagery after the BCI training was observed earlier (3). The shift of the activity to the same level during motor imagery of paretic and intact hands is regarded as a motor recovery index (40). The activity in SMA observed during the right and the left hand motor imagery can testify to the role of SMA in the controlling movements of the both arms (41).

The EEG analysis revealed four sources of brain electrical activity which were the most relevant to the BCI control. Two of the sources were typical for the EEG activity during motor imagery in both healthy subjects and patients with subcortical stroke (18). These sources were localized in the right primary somatosensory cortex (SIR) and precuneus (PRC), the areas unaffected by lesion. Two other sources (SILa and SILb) were specific for the patient in question in contrast to the symmetric SIL and SIR sources observed in healthy subjects and patients with subcortical stroke (18). These specific sources were localized at the posterior border of the damaged area, close to the central sulcus and approximately symmetric to SIR. We suppose that these sources represent the activity of the remaining parts of a damaged neural network in the primary somatosensory area of the left hemisphere symmetric to that observed in the intact (right) hemisphere. These sources demonstrate typical mu-rhythm desynchronization during the motor imagery, but they exhibit independent activity and their peak frequency is significantly lower than that of the similar sources of healthy subjects.

No task-specific EEG components were localized in SMA, while the SMA sources were expected (18) and the SMA activation was revealed by fMRI. This demonstrates the value of the integrated multi-modal analysis.

## Conclusions

Active use of hand motor imagery for controlling the exoskeleton by BCI is shown to contribute to positive dynamics of arm motor function recovery in a patient with extensive cortical lesion. Two BCI biomarkers corresponding to EEG activity in intact brain areas were similar to those observed earlier in healthy subjects and post-stroke patients with mild subcortical lesions. Another two biomarkers corresponded to the activity in primary somatosensory areas near the lesion edge. Their sources exhibited independent mu-rhythm activity with the peak frequency significantly lower than that of mu-rhythm in healthy subjects. Significant reduction in size of areas activated during the paretic hand movement imagery and increase for those activated during the intact hand movement imagery were observed by fMRI. The shift of the activations to the same level was regarded as the general index of the motor recovery. We conclude that the integrated analysis of EEG, fMRI, and motor activity allows to account for the reorganization of different levels of the motor system and to provide a comprehensive basis for adequate assessment of the BCI+ exoskeleton rehabilitation efficiency.

## Ethics Statement

The study was conducted in accordance with the Helsinki Declaration and was approved by the Ethical Committee of the Vladimirsky Moscow Regional Research Clinical Institute. The patient gave her written and informed consent to participate in the corresponding study. The patient gave her written and informed consent to publish the results of the study.

## Author Contributions

EB, AK, and ID performed the experiments and collected the data. EB, PB, and AS analyzed biomechanical, EEG, and fMRI data, respectively. AF, EB, PB, and JM contributed to the interpretation of the data and contributed to writing the manuscript. All authors approved the final version of the manuscript for submission.

### Conflict of Interest Statement

The authors declare that the research was conducted in the absence of any commercial or financial relationships that could be construed as a potential conflict of interest.
